# Mechanical effects in aging of the musculoskeletal system: Molecular signaling and spatial scale alterations

**DOI:** 10.1016/j.jot.2025.04.018

**Published:** 2025-05-27

**Authors:** Zeyuan Zhang, Fuming Cao, Dingfa Liang, Meng Pan, William W. Lu, Houchen Lyu, Yong Xie, Licheng Zhang, Peifu Tang

**Affiliations:** a Department of Orthopedics, The Fourth Medical Center, Chinese PLA General Hospital, Beijing, 100853, China; b State Key Laboratory of Complex, Severe, and Rare Diseases, Department of Immunology, Institute of Basic Medical Sciences Chinese Academy of Medical Sciences, School of Basic Medicine Peking Union Medical College, Beijing, 100005, China; c National Clinical Research Center for Orthopedics, Sports Medicine & Rehabilitation, Beijing, 100853, China; d Department of Orthopaedics and Traumatology, The University of Hong Kong, 999077, Hong Kong, China

**Keywords:** Aging, Musculoskeletal system, Mechanotransduction, Mesoscopic

## Abstract

The musculoskeletal system, the primary load-bearing structure of the human body, plays a crucial role in mechanotransduction, a process comprising mechanosensation, mechanotransduction, and mechanical effect. Aging leads to loss of ability of mechanosensitive cells to sense mechanical stimuli, disruption of transduction pathways, SASP and adiposity accumulation. At the mesoscopic level, bone, cartilage, and muscle differentiation decline, while adipogenesis increases, leading to extracellular matrix and structural aging, ultimately manifesting as macroscopic musculoskeletal degeneration. This review explores intercellular crosstalk and mechanotransduction alterations in aging from a mechanobiological perspective, providing insights into potential therapeutic targets for bone aging and osteoporosis. It also introduces the mesoscopic scale definition and trans mesoscopic transplantation therapy as novel strategies for fracture treatment, postoperative rehabilitation, and bone regeneration, offering innovative directions for future musculoskeletal research.

**The translational potential of this article:**

This article systematically reviews the effects of aging on the musculoskeletal system from a mechanobiological viewpoint, covering from microscopic molecular signaling to macroscopic spatial structural alterations, and proposes new strategies to complement the principles of AO therapy, optimization of braking, new insights into tumor metastasis and weight-bearing, and a new strategy for trans mesoscopic transplantation therapy. These insights will contribute to optimizing the management of geriatric fragility fractures in the elderly, exploring innovative therapies for the treatment of diseases of the aging musculoskeletal system, and facilitating the development of integrative therapies and precision medicine in the field of orthopaedics.

## Graphical abstract

**Figure:**
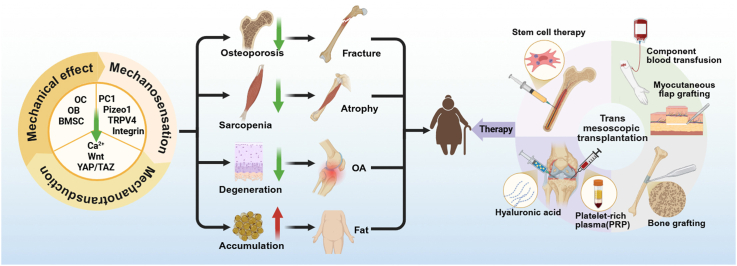

## 1 Introduction

Aging leads to the gradual decline of physiological functions across tissues, organs, and cells, particularly affecting the musculoskeletal system [1], which can result in osteoporosis and sarcopenia, increasing the risk of adverse health outcomes in the elderly [2]. Bones are constantly exposed to mechanical stimuli, such as gravity, fluid shear stress, and hydrostatic pressure [3].

These stimuli play a critical role in bone homeostasis, in elder adults, muscle function declines alongside skeletal deterioration, emphasizing the need to consider the skeleton and muscles as a cohesive system. Chondrocytes and adipocytes can also regulate cell growth and apoptosis by sensing mechanical stimuli as well as secreting some factors [4]. Therefore, bone, muscle, cartilage, and adipose tissue should receive equal attention when exploring mechanisms to slow bone aging.

Bone is mainly composed of osteocytes, osteoblasts, and osteoclasts. Osteocytes make up 90–95 % of the total cells in the adult skeleton and are the main mechanoreceptive cells. The response of osteocytes to mechanical stimuli includes three steps: mechanosensation, mechanotransduction and mechanical effect [5]. Disruptions in any of these processes can lead to bone loss and osteoporosis. The musculoskeletal system differs from other systems in that it is regulated by mechanical stimuli [6], making it crucial to explore aging mechanisms from a mechanosensitive perspective.

**Fig. 1:**
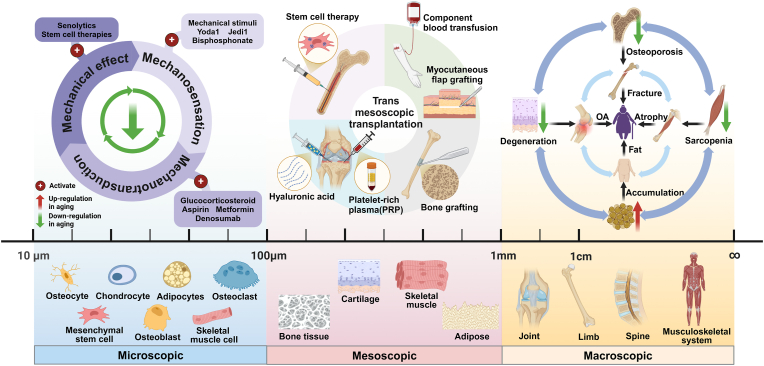
**Changes in the microscopic, mesoscopic and macroscopic structure of the musculoskeletal system in aging.** In the microscopic scale, mechanoreception, mechanotransduction, and mechanical effects are all impaired in aging, and the aging of the musculoskeletal system at the mechanical level can be attenuated by improving the three stages; in the mesoscopic scale, the trans mesoscopic scale transplantation therapies proposed in this review are widely used in clinical practice; and in the macroscopic scale, the simultaneous aging of bone, cartilage, muscle, and adipose tissues collectively results in the development of diseases of the aging musculoskeletal system.

In this review, we focus on crosstalk mechanisms of bone, muscle, cartilage and adipose tissue in bone aging explained from a mechanobiological perspective, with the aim of finding common therapeutic targets for slowing down aging in the musculoskeletal system and providing new therapeutic ideas for the design of surgical plans for fractures due to osteoporosis in elderly patients and how to perform postoperative rehabilitation (Fig. 1).

## 2 Mechanosenor

Sensing mechanical stimuli is the first step in the whole process of mechanotransduction, and varieties of cells in the musculoskeletal system rely on mechanosensitive receptors on the cell membrane to sense mechanical stimuli.

### 2.1 PC1

Polycystin-1(PC-1, encoded by Pkd1) is a typical mechanosensitive protein originally discovered on kidney cells [7], and plays a crucial role in osteomechanical sensing and maintenance of bone cell-mediated bone formation homeostasis [8].

#### 2.1.1 PC1 in osteocytes and osteoblasts

PC1 acts as mechanosensors in osteoblasts and osteocytes. The team of Xiao Z reported that conditional deletion of Pkd1 in osteocytes significantly attenuated mechanical load-induced bone formation [9]. Expression of PC1 in osteoblasts can lead to increased expression of runt-related transcription factor 2 (Runx2) and increased markers of osteoblast differentiation [10].

#### 2.1.2 PC1 in osteoclasts

PC1 has a different role in osteoclasts versus osteoblasts. PC1 levels in osteoclasts were elevated under mechanical unloading and microgravity conditions, so scholars believed that osteoclasts can sense mechanical unloading and mediate bone resorption through the PC1 pathway [11]. A recent study by Huang M et al. [12] demonstrated that osteoclasts can directly sense mechanical stimuli, and that PC1 facilitates nuclear translocation of transcriptional co-activator with PDZ-binding motifs (TAZ) through C-terminal tail cleavage, and promotes osteoclastogenesis and bone resorption.

#### 2.1.3 PC1 in other cells

PC1 can enhance the osteochondral differentiation potential of PSPC through nuclear translocation of TAZ [13]. Adipose deposition is a major feature of bone aging, and PC1 interacts with TAZ can not only stimulate TAZ-dependent osteoclastogenesis, but also inhibit adipogenesis [14]. PC1 activation causes TAZ to bind to PPARγ to inhibit adipose formation, which is stimulated when PC1 is deficient, leading to increased bone marrow adipose tissue (MAT) deposition and aggravated bone aging [15].

#### 2.1.4 PC1 and aging

Zhang T et al. [16] used gene-targeting mice to demonstrate that PKD1 plays a role in the background of senescence, and that PKD1 deficiency leads to increased sensitivity to reactive oxygen species (ROS), resulting in mitochondrial depolarization and ultimately apoptosis. Therefore, it is reasonable to hypothesize that the decreased expression of the Pkd1 gene with aging causes a decrease in PC1, which leads to decreased osteocyte sensitization to mechanical stimuli. These suggest that PC1 could serve as a potential new therapeutic target for the prevention of bone loss and aging.

### 2.2 PIEZO1

Piezo1 is a mechanosensitive ion channel on osteocytes that mediates the conversion of mechanical stimuli into biochemical signals. It is activated by mechanical loading, leading to Ca^2+^ influx and triggering downstream pathways that promote osteogenesis [17].

#### 2.2.1 PIEZO1 in osteocytes and osteoblasts

Like PC1, Piezo1 produces different effects when expressed on different cell types. Piezo1 deficiency in osteoblasts leads to bone loss, spontaneous fracture and increasing bone resorption. Hu Y et al. [18] studied the effect of the Piezo1 on bone loss in a microgravity environment and found that impaired Piezo1 function led to insufficient bone formation. Piezo1 can also promote osteogenesis through the YAP/β-catenin signaling axis and significantly accelerate the bone reconstruction in the defect area [19], which provides a new direction for the diagnosis and treatment of osteoporosis and bone aging.

#### 2.2.2 PIEZO1 in osteoclasts

Shen Y et al. [20] found that increased Piezo1 expression was accompanied by significant increases in RANKL and OPG expression. Piezo1 regulates the activation of the NF-κB pathway and promotes osteoclastogenesis [21]. However, Piezo1 deficiency in osteoclasts does not affect osteogenesis and resists bone loss induced by mechanical unloading.

#### 2.2.3 PIEZO1 in other cells

Piezo1 activation promotes proliferation and osteogenic differentiation of bone marrow mesenchymal stem cells (BMSCs) through the β-catenin pathway [18]. Piezo1 is stably expressed not only in the musculoskeletal system, but also in cartilage, tendons, intervertebral discs, and even their surrounding connective tissues [22]. Yoda1, an activator of PIEZO1, promotes osteoblast differentiation and inhibits adipocyte differentiation [23].

#### 2.2.4 PIEZO1 and aging

Li X et al. found that Piezo1 expression decreases with age in mice, leading to reduced bone responsiveness to mechanical loading and increased cortical bone loss [24]. Piezo1 deficiency in osteoblasts and osteocytes exacerbates age-related bone loss. Abnormal Piezo1 activation can also promote chondrocyte and disc senescence [25,26]. Ma et al. [27] showed that Piezo1 enhances iron overload by increasing erythrocyte turnover, contributing to musculoskeletal aging. Piezo1 is associated with vascular senescence, nervous system demyelination and neurodegeneration [28]. Exploring changes in Piezo1 with aging and identifying therapeutic targets is key to preventing bone aging.

### 2.3 TRPV4

The transient receptor potential vanilloid 4 (TRPV4) is a mechanoreceptor expressed in areas of high mechanical stress. When activated, it mediates Ca^2+^ influx, triggering downstream pathways that produce diverse effects depending on the stimulus [29].

#### 2.3.1 TRPV4 in osteocytes and osteoblasts

Lyons JS et al. [30] found that activated TRPV4 reduced sclerostin expression in osteocytes and promoted bone formation. TRPV4 plays an important role in osteoblasts' perception of hypotonic stimuli. In osteoblasts, TRPV4 and Piezo1 act synergistically to promote osteogenesis [31]. Activation of TRPV4 enhances intracellular Ca^2+^ levels and promotes osteoblast expression [32].

#### 2.3.2 TRPV4 in osteoclasts

At late stages of osteoclast differentiation, TRPV4 is a key mechanosensitive channel whose activation promotes osteoclast differentiation [33]. During osteoarthritis (OA) progression, hypotonic stimuli enhance TRPV4-mediated Ca^2+^ influx and induce RANKL mRNA expression, whereas knockdown of TRPV4 reduces osteoclast numbers and inhibits bone loss [34].

#### 2.3.3 TRPV4 in other cells

Khatib NS et al. demonstrated that TRPV4 is essential for bone and cartilage mechanotransduction and growth, and discovered the importance of TRPV4 in prenatal joint development [35]. O'Conor CJ et al. [36] found that TRPV4 can be protective in an obese OA model and that TRPV4 deficiency leads to accelerated joint degeneration with aging [36]. Subsequently, it was found that deletion of Trpv4 reduced the severity of aging-associated OA but did not alter the progression of OA [37].

#### 2.3.4 TRPV4 and aging

With aging, TRPV4 expression decreases, leading to reduced Ca^2+^ influx through the channel [38]. TRPV4-mediated Ca^2+^ influx also alleviates senescence in periodontal ligament stem cells (PDLSCs) [39]. Understanding TRPV4's crosstalk with other molecules and its age-related changes is crucial for advancing osteoporosis and bone aging therapies.

### 2.4 Integrin

Integrins are a class of transmembrane glycoprotein receptors consisting of non-covalent α/β heterodimers, with 18 types of α chains and 8 types of β chains binding to each other to form 24 different heterodimers. Integrins are important target proteins for mechanical stress stimulation on cells, and can convert extracellular physical and mechanical signals into intracellular bioelectric signals [40].

#### 2.4.1 Integrin in osteocytes and osteoblasts

Integrin α5β1 is highly expressed in osteocytes and osteoblasts. Mechanical stimuli upregulate the α5 subunit, enhancing osteogenesis and bone repair. Marie PJ et al. [41] showed that a peptidomimetic ligand of integrin α4β1 promoted osteoblast differentiation and increased bone mass in osteopenic mice. Upregulation of integrin β1 inhibits osteoblast apoptosis and enhances osteoblast migration and fracture healing [42]. Deletion of the α5 subunit in osteocytes reduces bone formation, while deletion of the β3 subunit impairs osteoblast differentiation and bone formation [43].

#### 2.4.2 Integrin in osteoclasts

Integrin αvβ3, a key signaling molecule involved in the formation of the osteoclast cytoskeleton, is abundantly expressed on osteoclasts [44]. Integrin β1 is associated with breast cancer cell proliferation and osteoclast maturation [45]. In addition, increased matrix stiffness can also promote osteoclastogenesis by inhibiting the integrin β3-mediated mechanotransduction process [46]. Inhibition of αvβ3 function on osteoclasts is now becoming a therapeutic option for targeting osteoporosis and bone metastasis.

#### 2.4.3 Integrin in other cells

Integrin α1β1 plays a crucial role in chondrocyte transduction in response to hypo-osmotic stimuli, and deletion of the α1 subunit can inhibit chondrocyte activation of TRPV4 and response to hypo-osmotic stimuli [47]. Macrophages can increase osteogenesis in BMSCs by triggering integrin αVβ3 and secreting TGF-β to enhance bone regeneration [48]. Deficiency of Integrin β1 also enhances adipogenic differentiation of BMSCs.

#### 2.4.4 Integrin and aging

Integrin β3 subunit identified as both a marker and regulator of aging [49]. Age-related declines in the protein complexes localized for focal adhesion reduce focal adhesion turnover, causing abnormal integrin signaling, ROS production, and accelerated aging [50]. Activation of integrin β4 is linked to cellular senescence, with α6β4 activation in irradiated cancer cells inducing premature senescence [51].

## 3 Mechanotransduction

Mechanotransduction is the process by which mechanosensitive ion channels or receptors on the cell membrane sense biomechanical signals, triggering cytoskeletal changes and downstream biochemical signaling responses [52]. However, the impact of aging on this process remains underexplored.

### 3.1 Hippo-YAP/TAZ pathway

The Hippo signaling pathway regulates osteocyte proliferation, differentiation, and bone metabolism [53]. YAP and TAZ are key mechanosensitive regulators. When the Hippo pathway is activated, LATS1/2 phosphorylates YAP/TAZ, leading to their cytoplasmic retention and degradation. LATS1/2 deficiency enhances YAP nuclear translocation, promoting osteogenic differentiation and proliferation [54].

#### 3.1.1 YAP/TAZ in osteocytes and osteoblasts

YAP/TAZ exhibit different expression levels and functions across osteocyte stages. Inhibiting the Hippo pathway promotes osteogenic differentiation. YAP/TAZ suppress osteoblast progenitor differentiation but enhance bone formation in mature osteoblasts while inhibiting bone resorption. Gargalionis AN et al. [55] found that TAZ enhances RunX2 activity, promoting fracture healing and alleviating delayed union caused by mechanical unloading.

#### 3.1.2 YAP/TAZ in osteoclasts

YAP/TAZ inhibit osteoclasts, and TAZ knockdown increases osteoclast formation, leading to bone resorption. Dephosphorylated YAP/TAZ block RANKL signaling directly or by enhancing OPG expression. Additionally, the PC1-TAZ axis forms a mechanotransduction complex, making it a potential therapeutic target for osteoporosis, bone aging, and related conditions [56].

#### 3.1.3 YAP/TAZ in other cells

YAP co-expresses with β-catenin in BMSCs, promoting osteogenesis and inhibiting adipogenesis. YAP/TAZ regulate transcription factors like Runx2 and Sox9, playing crucial roles in cartilage development. YAP promotes chondrogenic progenitor proliferation, while TAZ has an opposing role in chondrocyte maturation. YAP/TAZ deficiency leads to chondrogenic dysplasia and cartilage damage [57].

#### 3.1.4 Hippo-YAP/TAZ and aging

The Hippo pathway is influenced by aging factors such as mechanical stress and mitochondrial dysfunction. Aging reduces mechanotransduction and downregulates YAP/TAZ expression. Studies show YAP/TAZ levels peak in young mice but decline with age [58]. Aging-induced Hippo pathway activation increases YAP phosphorylation, preventing nuclear translocation. Further exploration of aging-related Hippo regulation may reveal new therapeutic targets.

### 3.2 Wnt/β-catenin

Wnt is a secreted lipid-modified protein that binds to different receptors on the cell membrane. The Wnt signaling pathway is a key regulator of bone metabolism, particularly in bone formation. The canonical Wnt pathway stabilizes intracellular β-catenin under mechanical loading by binding to low-density lipoprotein receptor-associated protein (LRP) and frizzled (FZD) co-receptors, thereby regulating gene transcription [59].

#### 3.2.1 Wnt/β-catenin in osteocytes and osteoblasts

The Wnt/β-catenin pathway enhances osteoblast differentiation, promotes bone formation, and inhibits osteoblast apoptosis, leading to increased bone mass and density [60]. It directly affects osteoblasts and osteocytes by stimulating bone formation and reducing resorption. Wnt7b expression increases in aging bones and during fracture healing, significantly boosting bone mass, making it a potential target for therapeutic intervention [61].

#### 3.2.2 Wnt/β-catenin in osteoclasts

Wnt signaling inhibits osteoclastogenesis through the canonical pathways of up-regulation of OPG and down-regulation of RANKL, whereas noncanonical Wnt signaling in osteoclast precursors enhances RANKL-induced osteoclastogenesis through Ror2-mediated non-canonical Wnt signaling [62].

#### 3.2.3 Wnt/β-catenin in other cells

Wnt/β-catenin signaling promotes BMSC differentiation into osteoblast precursors, regulates the Runx family, and influences chondrogenesis, muscle development, and adipogenesis. mRNA-encoded β-catenin activation in chondrocytes facilitates cartilage-to-bone transformation and accelerates fracture repair [63]. However, excessive Wnt signaling in aged myogenic progenitor cells shifts muscle stem cells toward fibrosis, accelerating muscle aging [64].

#### 3.2.4 Wnt/β-catenin and aging

The effects of aging on Wnt/β-catenin signaling vary across tissues. Studies show a decline in Wnt-related gene expression in aging mice, and β-catenin levels decrease in senescent BMSCs, with chronic Wnt stimulation potentially leading to stem cell depletion [65]. In aged human arterial cells, Wnt signaling is activated but fails to induce proliferation. Due to its dual role in promoting bone formation and inhibiting resorption, Wnt/β-catenin signaling may serve as a potential therapeutic target for preventing bone aging.

### 3.3 Ca^2+^

Ca^2+^ (Calcium) plays a crucial role in the body, acting as a key signaling molecule in osteocytes, neurons, and immune cells, and is essential for maintaining bone homeostasis [66]. As a major bone component, Ca^2+^ forms hydroxyapatite crystals within osteoblast matrix vesicles and participates in mechanical signal transmission [67].

#### 3.3.1 Ca^2+^ in mechanotransduction

Ca^2+^ serves as a downstream signaling molecule for mechanosensors like Piezo1, Polycystins, and TRPV. Upon mechanical stimulation, these sensors mediate Ca^2+^ influx, activating pathways such as CaM, PI3K, and PKC [68], which regulate osteogenesis through Runx2, Wnt/β-catenin, YAP/TAZ, and ERK signaling. In chondrocytes, Piezo1, Piezo2, and TRPV4 channels facilitate Ca^2+^-mediated mechanotransduction, influencing apoptosis and osteogenic responses [69].

#### 3.3.2 Ca^2+^ and aging

Aging leads to bone mass loss and calcium depletion, impairing osteocyte Ca^2+^ signaling and reducing mechanical responsiveness. Piezo1 inactivation in muscle stem cells elevates Ca^2+^ influx and ROS levels, accelerating senescence and reducing mechanosignaling. Ca^2+^ accumulation due to aging exacerbates cellular damage via ROS-induced mitochondrial dysfunction. However, elevated Ca^2+^ can activate Cx43 hemichannels, protecting osteocytes [70].

## 4 Effector cell

Osteocytes, osteoblasts, osteoclasts, chondrocytes, skeletal muscle cells, and adipocytes are key effectors of the musculoskeletal system in response to mechanical stimuli. Aging, mechanical unloading, and other factors upregulate aging-related factors, downregulate mechanotransduction, and reduce the ability of bone to respond to mechanical loading, leading to microstructural changes, bone loss, osteoporosis, and skeletal aging. Understanding the regulation of these effector cells is critical to addressing osteoporosis and bone aging.

### 4.1 Osteocytes and osteoblasts

#### 4.1.1 Osteocytes in aging

Osteocytes are key mechanosensitive cells that play a crucial role in maintaining bone homeostasis and regulating the skeletal response to mechanical stimuli. Research by Tatsumi S et al. [71] demonstrated that osteocyte-ablated mice exhibit fragile skeletons, intracortical porosity, osteoclast dysfunction, trabecular bone loss, deteriorated microstructure, and adipose tissue accumulation in bone marrow, all hallmarks of bone aging. These cells, embedded in the bone matrix, form an extensive network through dendrites that allows for efficient mechanosensing and transduction. Normal osteocyte morphology supports mechanosensitivity, but excessive mechanical stimuli or mechanical unloading disrupts mechanotransduction [72]. Aging osteocytes also exhibit morphological changes, such as alterations in their luminal structures, which further contribute to bone aging characterized by impaired mechanosensitivity, ROS accumulation, and dysfunction in network remodeling [73].

#### 4.1.2 Osteoblasts in aging

Osteoblasts, responsible for bone formation, are critical for bone growth and maintenance, but aging leads to their dysfunction [74]. Mechanical stimuli enhance osteogenesis more than bone resorption, highlighting osteoblasts’ crucial role in mechanically driven bone formation. Aging impairs osteoblast mineralization, and osteoblast apoptosis triggers a pro-inflammatory response, leading to progressive bone loss [75]. Senescent osteocytes and their secreted senescence-associated secretory phenotype (SASP), along with osteokines, may further contribute to age-related bone loss, with SASP markers being more prominently expressed in osteoblastic progenitor cells and osteoclasts [76].

### 4.2 Osteoclasts

Osteoclasts are the only cells in bone tissue capable of resorbing bone and play an important role in bone aging, repair, and sensing mechanical loads.

#### 4.2.1 Osteoclasts in aging

Osteoclasts undergo significant changes with aging, particularly in their ability to sense mechanical stimuli, leading to increased activity and bone loss. Farr JN et al. found that senescent cell-conditioned medium enhances osteoclast progenitor survival, promoting osteoclastogenesis [77]. Senescent skeletal stem cells (SSCS) also have an enhanced ability to stimulate osteoclast formation [78], and SASP accelerates osteoclast differentiation, contributing to osteoporosis. Aging causes microdamage in cortical bone from increased intracortical resorption, roughening the bone surface and increasing resorption pits, indicating enhanced osteoclast activity [79]. In postmenopausal women, decreased estrogen levels exacerbate bone loss by reducing DNA methylation and generating more aggressive osteoclasts [80]. Interestingly, eliminating senescent osteoclast progenitors has little effect on age-related bone loss, suggesting that other senescent cells, such as osteoblasts or mature osteoclasts, may be central to the aging process.

### 4.3 Bone marrow mesenchymal stem cells (BMSCs)

BMSCs are pluripotent progenitor cells with self-renewal and multidirectional differentiation capabilities. They can differentiate into osteoblasts, chondrocytes, skeletal muscle cells, and adipocytes, and play a crucial role in maintaining bone mechanosensitivity. Mechanical loading affects BMSC proliferation and differentiation into osteogenic, chondrogenic, or adipogenic lineages [81].

Aging BMSCs show reduced osteogenic potential and high levels of pro-inflammatory cytokines promoting osteoclast activity [82]. They also induce adipogenic differentiation via lncRNA NEAT1, contributing to bone loss. BMSC senescence is influenced by DNA damage, oxidative stress, and factors like hormones, immune responses, and signaling molecules (p53, Akt, STAT3, etc.). Specific miRNAs regulate the balance between osteogenesis and adipogenesis. Senescent osteocytes and their SASPs can alter BMSC differentiation, further promoting bone loss.

#### 4.3.1 Chondrocytes in aging

Chondrocytes, mechanosensitive cells derived from MSCs, respond to mechanical stimuli during mechanotransduction. Chondrocyte numbers decline with aging, influenced by factors like ROS, mitochondrial autophagy, and Piezo1 modulation. Physiological mechanical loads promote cartilage growth, while excessive stimuli activate Akt signaling, leading to ROS accumulation and chondrocyte senescence. Age-related mitochondrial dysfunction and oxidative stress increase SASP, causing chondrocyte senescence [83]. Chondrocytes sense mechanical overload through PIEZO1, leading to ferroptosis and senescence via miR-155-5p expression and Ca^2+^ influx [84]. ECM changes, like increased stiffness, promote Klotho promoter methylation and accelerate senescence [85]. Chondrocyte senescence leads to OA, with cartilage destruction and reduced bone elasticity, increasing susceptibility to mechanical stress. Modulating mechanical signals to alleviate chondrocyte senescence is a promising OA treatment strategy.

#### 4.3.2 Skeletal muscle cells in aging

Skeletal muscle cells are mechanosensitive, responding to mechanical stimuli with contraction and myokine secretion. Piezo1 prevents senescence in muscle stem cells (MuSCs), promoting their proliferation and regeneration, but aging reduces mechanical signaling, increasing senescence and decreasing MuSCs numbers. Mechanical stimulation activates kinesin-1, enhancing satellite cell proliferation and muscle regeneration. The PI3K/Akt pathway mediates muscle loss in sarcopenia. Aging reduces mechanical signaling, leading to increased senescence and decreased MuSC numbers. Myokines, secreted by muscle cells, influence various functions, among which apelin declines with age, impairs muscle function [86]. Aging induces MuSC senescence, impairing regeneration and increasing apoptosis and mitochondrial dysfunction. It also reduces S-adenosylmethionine (SAM) and heterochromatin markers, leading to DNA damage and muscle weakness. Fat-infiltrated cells hinder muscle regeneration, and aging impairs neuromuscular junction integrity, increasing oxidative stress and inflammation [87]. Reduced mechanical activity accelerates muscle atrophy, contributing to sarcopenia, a progressive loss of muscle mass and strength. Regular physical activity enhances muscle strength and bone mass, delaying musculoskeletal aging.

#### 4.3.3 Adipocytes in aging

Adipose tissue, consisting of adipocytes derived from BMSCs, influences bone metabolism and cartilage growth through adipokines like leptin and adiponectin, which regulate bone formation and serve as osteoporosis biomarkers. Aging and obesity increase inflammatory adipokines, creating a pro-inflammatory microenvironment that affects bone health [88]. Mechanical stimulation can modulate BMSC differentiation, promoting osteogenesis while inhibiting adipogenesis. As aging progresses, BMSCs show reduced osteogenesis and increased adipogenesis, leading to MAT accumulation and osteoporosis. MAT accumulation is linked to osteoclastogenesis and bone loss [89]. Adipose infiltration in bone and muscle accelerates aging, contributing to bone loss and muscle weakness. Mechanical signaling's role in reducing adipogenesis and improving bone quality is essential for treating osteoporosis.

**Fig. 2:**
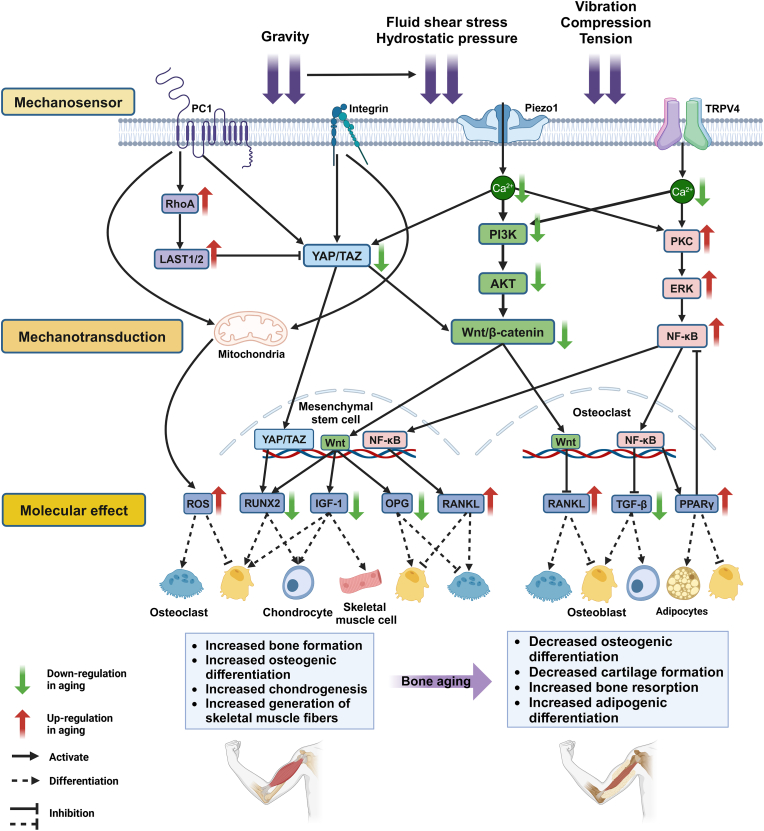
**Exploring the three components of mechanotransduction at the molecular, pathway, and cellular microscopic levels in aging.** This figure shows several mechanotransduction signaling pathways in terms of sensation, transduction, and effect, and labels the changes of each protein, signaling molecule, and effector molecule in aging, as well as the effects on various effector cells, with red arrows indicating up-regulation in senescence, and green arrows indicating down-regulation in aging.

Aging induces microstructural changes in the musculoskeletal system, impairing mechanoreception (Fig. 2). Mechanosensitive channels' number and sensitivity are reduced, and senescence signals, inflammatory factors, and negative pathways disrupt mechanotransduction. Restoring mechanosensitive channel activation and resolving these factors is key to maintaining effector cell activity.

## 5 Musculoskeletal mesoscopic structure

The microstructure of musculoskeletal system is a prerequisite for the integrity of musculoskeletal system growth, metabolism, and mechanosensitivity, and they play a key role in the mechanosensitive behavior of bone by sensing mechanical stimuli and coordinating the activities of various osteoblasts to participate in signaling for bone formation and bone resorption. With aging, this complex and dynamic process in the musculoskeletal system microstructure often becomes unbalanced, leading to increased bone resorption and insufficient bone formation, and ultimately to bone loss.

A mesoscopic structure is a scale structure between the microscopic and the macroscopic, a scale interval in which matter exhibits both micromechanical features and some macroscopic properties. It mainly involves the levels of bone trabeculae, bone units, and bone matrix in bones, and muscle fibers, cartilage matrix, and ECM in other organs and tissues of the musculoskeletal system [90]. Therefore, we suggest that the mesoscopic level of the musculoskeletal system is usually studied at scales of hundreds of micrometers to millimeters. Since the mesoscopic structure already has some functions at the macroscopic level, when the microstructure is altered, the mesoscopic and macroscopic structure and function are also altered, and we will describe for the alterations at the mesoscopic levels of the musculoskeletal system.

### 5.1 Bone mesoscopic structure

The strength and extensibility of bone is mainly due to the bone matrix, in which there exists a special kind of non-enzymatic cross-links, advanced glycation end-products (AGEs), which are formed by amino acid and glucose-mediated reactions in collagen, and bone aging can change the cross-linking characteristics. Progressive increase with aging, accumulation of AGEs reduces the mechanical properties of bone and the risk of fracture is higher [91]. In addition, aging leads to collagen fiber mineralization, resulting in lower fiber strain and loss of fracture resistance in aging bone. Age-related changes in bone are characterized by a decrease in the number of trabeculae, trabecular structural voids, and an increase in cortical porosity. During bone aging, aging-related signals in BMSCs are activated, and the osteogenic potential, hemotransplantation rate, and rate of cellular repair of BMSCs gradually decrease, and the adipogenic capacity is elevated, leading to a subsequent decrease in cortical bone size, trabecular volume, and bone strength. Of these, more cortical bone loss occurs at peripheral sites than trabecular bone loss, and increased cortical porosity is associated with decreased bone strength, which is consistent with the epidemiologic evidence that the majority of fractures in the elderly occur predominantly at the cortical periphery [92].

### 5.2 Cartilage mesoscopic

The mesoscopic structure of cartilage includes the perichondral matrix, extrachondral matrix, and so on. Biomechanical dysfunction at the mesoscopic scale due to aging is both a result of micro-scale (molecular) dysfunction and further exacerbates or causes new micro-scale dysfunction.

The physical properties of cartilage are determined by the composition of the extracellular matrix (ECM), which has significant shock-absorbing properties of its own, while the hydrostatic pressure generated by its impediment to fluid movement between tissues protects the tissues from compressive forces. ECM sclerosis is a typical feature of cartilage aging. sclerosis of the ECM initiates pathogenic mechanotransduction signals and impairs chondrocyte function, disrupting the balance between ECM synthesis and catabolism and accelerating the course of OA. The mismatch between pericellular matrix (PCM) and ECM amplified chondrocyte compressive strain, and that changes in PCM properties may alter the stress–strain and fluid flow environment of chondrocytes. Aging cartilage tissue produces more matrix metalloproteinases, and these proteases may lead to the degradation of PCM [93], and lead to the disturbance of the biomechanical environment of chondrocytes, which in turn leads to macrostructural damage and aggravates the progression of OA.

### 5.3 Muscle mesoscopic structure

Senescence negatively affects the overall multicellular crosstalk as well as the relative cell number in skeletal muscle and reduces the regenerative capacity of BMSCs [94]. In the aging environment, skeletal muscle cells with reduced mechanical sensitivity are subjected to a biochemical environment of attenuated mechanical stimuli and various aging factors, which can lead to a significant decrease in the number, strength, extensibility and elasticity of muscle fibers. Human skeletal muscle atrophy seems to be inevitable with age. The loss of muscle fibers begins at about 50 years of age and continues until 80 years of age.

In the context of muscle aging, it is not just the loss of muscle mass that leads to deterioration of muscle function. Other factors that support muscle mass also come into play, including muscle composition, metabolism, fat penetration, insulin resistance, fibrosis, etc. For example, in aging muscle, cilia on MuSC undergo loss with reduced regenerative capacity, which reduces muscle repair capacity [95].

### 5.4 Adipose mesoscopic structure

**Fig. 3:**
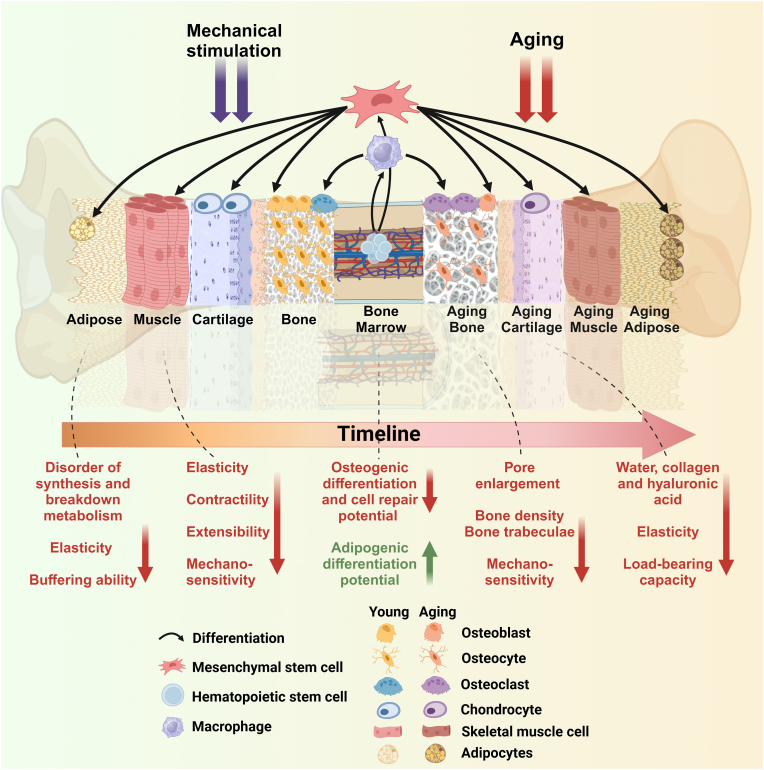
**Exploring changes in the mesoscopic and macroscopic structure of the musculoskeletal system in aging.** This figure shows the changes in the different tissues of the musculoskeletal system in aging in the macroscopic dimension. The left half is the young skeleton and the right half is the aging skeleton. As various types of cells change in the aging environment, the properties and structures of the aging bone, muscle, cartilage, adipose, and other tissues change, leading to the overall aging of the musculoskeletal system, resulting in bone loss, sarcopenia, osteoarthritis, obesity, and other aging-related diseases.

The mesoscopic structure in fat consists of fat lobules, fat matrix, and various fibers. Fibrosis of the adipose matrix disrupts the normal distribution of forces in the joints during exercise, and Béatrice Dirat et al. [96] found that preconditioning the extracellular matrix and stromal vascular fraction of the adipose tissue could have a positive effect. Age affects the characterization of infrapatellar fat pad (IFP) and is associated with the development of OA, and adiposity can regulate cellular senescence by modulating matrix metalloproteinase-2 [97]. Collagen deposition and extracellular matrix remodeling in adipose tissue contribute to sarcopenia. Therefore, restoring the mesoscopic structural viability of adipose, such as autologous adipose grafting and platelet-rich plasma (PRP) therapy may be the future direction of regenerative medicine. When changes occur in microstructures such as molecules and cells, changes also occur at the meso- and macroscopic levels, which can lead to changes in tissue structure and even at a more macroscopic level to the development of certain diseases (Fig. 3).

## 6 Discussion

Bone is a highly dynamic and metabolically active tissue that continuously senses and responds to mechanical stimuli throughout life, undergoing constant remodeling to maintain its robust structure and function. The main mechanical studies at the cellular level are cellular biomechanics and mechanobiology. Mechanobiology is a new doctrine that has rapidly emerged in recent years, focusing on the effects of force on cells or tissues, and how cells use force to carry out biological behaviors, so we prefer to use mechanobiology to provide an explanation of the behavior of bone cells that sense mechanical stimuli.

Appropriate mechanical stimuli are crucial for bone growth and function, as they are closely linked to mechanical loading. Skeletal response to mechanical stimuli involves perception, signal transduction, and the actions of various cells like osteocytes, chondrocytes, muscle cells, and adipocytes. Many studies have explored the roles of these mechanotransduction components in the musculoskeletal system (Table .1). This review focuses on how aging alters these processes and affects skeletal mechanosensitivity.

**Table 1:** Summary of previous studies on the role of mechanosensitive pathways.

Mechanosensor	Cell type/tissue	Mode of stimulation	Downstream pathways and factors	Function	Changes with aging/Relationship with aging	Species	Study design	Reference
PC1	Osteoblasts and osteocytes	Fluid shear stress	/	Conditional deletion of Pkd1 in osteocytes disrupts skeletal mechanosensing in mice	/	Mouse	In vivo	[9]
PC1	Osteoblasts and osteocytes	/	Runx2	polycystin-1 in osteoblasts/osteocytes is associated with skeletogenesis and Runx2 expression	/	Mouse	In vivo	[10]
PC1	Osteoclasts	Mechanical loading	TAZ	PC1 directly regulates osteoclastogenesis and bone resorption	/	Human and mouse	In vivo	[12]
PC1	Osteoblasts	Mechanical stimulation	TAZ	PC1 stimulate osteoblastogenesis and inhibit adipogenesis	/	Mouse	In vivo	[14]
Piezo1	Chondrocytes	Mechanical stimulation	Ptgs2 and Ccn2	Piezo1 is not only physiologically required for endochondral ossification, but also for pathological processes linked to OA progression and osteophyte formation.	/	Mouse	In vivo	[4]
Piezo1	PDLCs/Osteoblasts and osteoclasts	Compressive force	β-catenin	Activatesβ-catenin to regulate alveolar bone remodeling, increased osteogenic differentiation	/	Human	In vivo	[19]
Piezo1	Osteoblasts and osteocytes	Mechanical stimulation	Tnfrsf11b (encoding anti-osteoclastogenic protein OPG)	Protecting against age-associated cortical bone loss by inhibiting bone resorption	Piezo1 expression declines with age	Mouse	In vivo	[24]
Piezo1	Chondrocytes	Mechanical stress	p38MAPK and NF-κb activate IL-6 and IL-1β	Piezo1 sensed mechanical stress and promoted chondrocyte senescence via its Ca^2+^ channel ability	/	Human	In vitro	[25]
TRPV4	Osteocytes	Fluid shear stress	Ca^2+^	Microtubules tune mechanotransduction through TRPV4 to decrease sclerostin abundance in osteocytes	/	Mouse	In vivo	[30]
TRPV4	Osteoblasts	Mechanical stimulation	Ca^2+^	TRPV4 regulates osteoblast differentiation	/	Mouse	In vitro	[32]
TRPV4	Osteoclasts	/	Ca^2+^ and NFATc1	TRPV4-Mediated Calcium Influx Regulates Terminal Differentiation of Osteoclasts	/	Mouse	In vivo	[34]
TRPV4	Bone marrow-derived stem cells(BMSCs) and adipose-derived stem cells(ASCs)	/	/	Increased susceptibility of Trpv4-Deficient Mice to Obesity and Obesity-Induced Osteoarthritis with Very High-Fat Diet	/	Mouse	In vivo	[36]
TRPV4	Chondrocytes	/	Ca^2+^	Cartilage-specific knockout of TRPV4 decreases age-related osteoarthritis	Knockout of TRPV4 decreases age-related osteoarthritis	Mouse	In vivo	[37]
Integrin	Osteoblasts	/	/	BDNF promoted osteoblast migration and fracture healing by up-regulating integrin β1	/	Mouse	In vivo	[42]
Integrin	Osteocytes	Load-bearing	/	Osteocyte β3 integrin promotes bone mass accrual and force-induced bone formation in mice	/	Mouse	In vivo	[43]
Integrin	Osteocytes	Compressive force	Cx43 and PGE2	Osteocytes regulate bone anabolic response to mechanical loading in male mice via activation of integrin α5	/	Mouse	In vivo	[44]
Integrin	Osteoclasts	Mechanical stimulation	RhoA-ROCK2-YAP	Matrix stiffness regulates osteoclast fate through integrin-dependent mechanotransduction	/	Mouse	In vitro	[46]
Integrin	Chondrocytes	/	/	Blockage of Osteopontin-Integrin β3 Signaling in Infrapatellar Fat Pad Attenuates Osteoarthritis in Mice	/	Mouse	In vivo	[49]

### 6.1 Definition of the mesoscopic scale of the musculoskeletal system

This review describes the various types of mechanosensitive proteins, the signaling molecules involved in mechanotransduction, and the various types of cells that produce the effects from mechanosensation to mechano-effects, which we define as microscale alterations, and at the macroscale level we describe the alterations that occur in the aging state of bone, muscle, cartilage, and adipose, as well as the clinical disorders that they cause. However, between microscopic and macroscopic, there exists a mesoscopic scale [98], and in the musculoskeletal system this concept has not been clearly defined, so this review explains the mesoscopic scale component in the musculoskeletal system. From the biological-functional point of view, the structure formed by groups of cells with similar morphology and structure and close function, combined by intercellular matrix, is called tissue. Similar to the definition of tissue, in the spatial structural scale, we refer to such components that both contain micro-scale cellular structures and interactions and are able to mimic macro-scale organ functions and physiological responses as mesostructures. Firstly, all kinds of extracellular matrix, such as bone matrix, cartilage matrix, etc., all belong to the range of mesoscopic scale, in the skeleton, mesoscopic structure also mainly includes bone trabeculae, cortical bone and cancellous bone, etc.; in the muscle, including muscle fibers, muscle bundles; in the adipose tissue, including fat leaflets, and all kinds of elastic fibers, collagen fibers composed of fat matrix, etc. We define and elucidate the issue of mesoscopic scaling in the musculoskeletal system, with the expectation of generating interest and attention to mesoscopic scaling to further expand the depth and breadth of understanding in the prevention and treatment of aging in the musculoskeletal system.

### 6.2 Classification of mechanical stimuli

Osteocytes can perceive various mechanical stimuli, including gravity, fluid shear stress, hydrostatic pressure, vibration, compression, and tension. Fluid shear stress is a significant force that opens mechanosensitive ion channels. The shear stress generated by interstitial fluid flow (IFF) in the bone mesenchyme modulates osteoblast activity and provides mechanical stimuli to osteocytes. Bone deformation due to mechanical stress alters the fluid pressure gradient, driving interstitial fluid flow that impacts osteocytes. Hydrostatic pressure from physiological states also positively affects bone formation. Gravity is essential for bone health, and changes in gravity, such as in microgravity, can reduce interstitial fluid flow and hydrostatic pressure, affecting osteoclast formation. Microgravity environments, like space travel, lead to severe bone loss, and impaired osteocyte mechanosensitivity may accelerate bone aging, even with exercise countermeasures [99]. Oscillatory forces, such as ultrasound, promote fracture healing by generating mechanical stimuli and increasing blood flow, while compression enhances blood perfusion to support bone growth. Tension promotes osteogenic differentiation of BMSCs and osteoblast proliferation.

In summary, mechanical stimuli impact osteocyte homeostasis by altering matrix morphology and fluid shear stress, activating mechanosensitive ion channels, and prompting cellular responses to mechanical forces.

### 6.3 Restoration of impaired mechanical response processes

#### 6.3.1 Activation of impaired mechanosensitivity

Bone aging can lead to decreased mechanosensation. How to improve the mechanosensitivity of senescent cells is one of the ways to combat bone aging, and the activation of mechanosensitive channels can be achieved by mechanical stimuli and the intervention of certain drugs. Haitao Guan et al. prepared ZOL-PLGA@Yoda1/SPIO using the Piezo1 activator Yoda1 and found that ZOL-PLGA@Yoda1/SPIO activated Piezo1 in the bone defect region of osteoporotic mice and improved osteogenesis through the YAP/β-catenin signalling axis without significant side effects [100]. Integrin-binding mimetic peptides enhance osteoblast adhesion, proliferation and differentiation on titanium, and combining specific integrin-binding peptides with osteogenic peptides on orthopaedic implants produces positive results. Kang H et al. [101] found that titania nanotubes (Ti-NTs) can inhibit osteoclastogenesis and promote osteogenesis by inhibiting integrin ανβ3 in osteoclast precursor cells. Cilengitide and Tablysin-15 significantly inhibits osteoclastogenesis and improves osteoporosis by binding to ανβ3 [102].

#### 6.3.2 Restoring impaired mechanotransduction

Bone aging can lead to decreased mechanotransduction. Increasing Ca^2+^ levels in cells, inhibiting over-activated Hippo and NF-κB pathways as well as activating Wnt/β-catenin signalling are future directions of research to address the down-regulation of mechanotransduction. Calcium supplementation not only increases Ca^2+^ levels in signal transduction and upregulates the activation of various osteogenic signals, but also provides raw material for bone formation. Glucocorticoids, metformin [103], denosumab, and aspirin can inhibit the NF-κB pathway, and the inhibitory effect of Chinese medicine on the NF-κB pathway has recently become a hot topic of research. These therapeutic regimens can mitigate bone aging while reducing the inflammatory response, and they can alleviate bone loss from multiple targets of action.

#### 6.3.3 Restoration of damaged mechanical effects

Bone aging leads to reduced mechanical effects, making the removal of senescent cells or replenishment of young stem cells a promising treatment for bone aging and osteoporosis. Senolytics, a well-established anti-aging therapy, targets senescent cells and has shown potential in reversing aging in various tissues [104]. A systematic review by Novais EJ et al. concluded that senolytics, such as the combination of Dasatinib and Quercetin, can effectively slow bone aging, reduce inflammation, and mitigate age-related bone diseases [105]. Additionally, small extracellular vesicles (sEVs) with bone-targeting peptides can selectively eliminate senescent bone cells.

Hyaluronic acid hydrogel microspheres (AHM) enriched with pro-apoptotic liposomes (A-Lipo) can induce apoptosis in senescent chondrocytes, protecting normal chondrocytes and maintaining the chondrogenic differentiation ability of BMSCs [106]. Stem cell supplementation is another approach to reduce cellular senescence and lower inflammatory markers in aging individuals. The development of senolytic drugs and stem cell therapies presents a promising intervention for promoting bone health and preventing bone-related diseases.

### 6.4 Inspiration of clinical issues

#### 6.4.1 Transplantation across mesoscopic scales

**Fig. 4:**
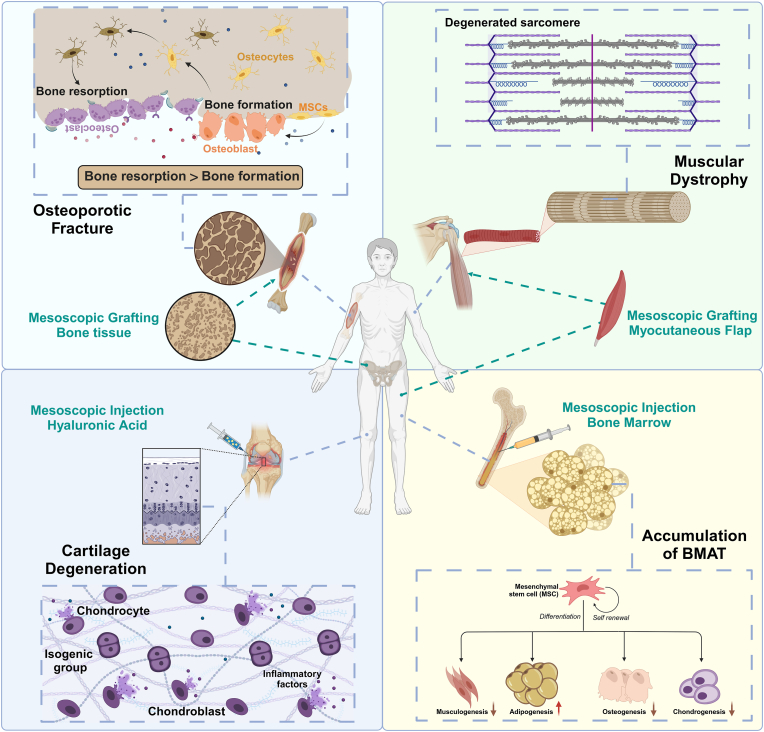
**Trans mesoscopic scale transplantation therapies for aging-related musculoskeletal disorders.** This figure shows the changes in aging from cells to tissues, which lead to the emergence of different pathological states and the occurrence of various diseases, and the combined effect of which results in the frequent occurrence of diseases related to aging of the musculoskeletal system in the elderly population. The application of trans mesoscopic scale transplantation therapies can alleviate clinical symptoms and reduce the progression of the diseases of aging.

Mesoscopic scale characterization and its cross-dimensional relevance are the focus of contemporary interdisciplinary research. According to our above definition of mesoscopic scale of musculoskeletal system, mesoscopic is a certain class of components that exists between cells and organs with certain functions but can not be called organs, besides musculoskeletal system, other tissues and organs of the human body contain many mesoscopic structures, such as nerve fiber bundles, plasma, and vascular connective tissues. The mesoscopic as a measure between micro and macro structures is often overlooked, even when we use it. Many scenarios involve the transfer of mesoscopic structures, such as bone grafts, hyaluronic acid injections, PRP, lipofilling therapy, myocutaneous flap transplantation, and component blood transfusions (Fig. 4). In clinical practice, most bone grafts are made from autogenous or allogeneic bone, and the ilium is often used as a site for bone grafting because of its special location and rich content of BMSCs, indicating that the mesoscopic structure should have a good ability to differentiate in order to meet the transplantation requirements. The mesostructures of different organs can work together, bone grafting combined with PRP to treat necrosis of the femoral head and were able to achieve good efficacy [107]. In recent years, although controversial, mesenchymal stromal cells have also been used to treat graft-versus-host disease with exciting results, reflecting the vast promise of transplantation across mesenchymal structures. An in-depth understanding of mesoscopic structure can enhance research on current stem cell and tissue therapy products for effective regulation [108]. Mesoscale science is a discipline that has the potential to fundamentally update the existing structure of knowledge, which differs from traditional models of unit-scale and system-scale research and reveals previously inaccessible areas of scientific research [109].

#### 6.4.2 New insights into AO treatment principles

Through in-depth study of the mechanosensitive perspective, we found new insights and interpretations of many issues in the clinic. The treatment principles proposed for AO include: restoration of anatomy, stable fracture fixation, preservation of blood supply, and early mobilization of the limb and patient, which emphasise anatomical repositioning and take into account biology and biomechanics, and have been used by clinicians up to the present day, whereas we provide a new interpretation of the treatment principles of AO from the mechanosensitive perspective and raise new questions.

The first principle of AO is anatomical repositioning. From the mechanical point of view, anatomical repositioning is to restore the natural line of force of the body. Humans are the only creatures in nature that walk upright, and the line of force of the body is particularly important, which is the key to maintaining the bones to feel the stress stimulation. When the natural alignment is restored, the fracture broken ends can feel the appropriate mechanical stimulation, which promotes the osteogenic differentiation, and accelerates the healing of the fracture. The fracture can be healed faster by promoting osteogenic differentiation. Strong fixation is the basis for early mobility, which is also aimed at enabling the bones to feel mechanical stimulation to accelerate fracture healing and to reduce stiffness at the fracture site, especially in the joints.

In addition, we raise new issues that need to be discussed in the principles of AO, i.e. whether they are equally applicable to the aging population, whether anatomical repositioning can satisfy the stress stimulation required by the aging skeleton, whether the osteoporotic bone in the aging population can satisfy the need for strong fixation, and the paradox of the need for immobilization versus early mobilisation after internal fixation.

#### 6.4.3 Fixation, immobilization and motion

Fracture healing requires both appropriate mechanical and biological conditions. Mechanical loading is essential for bone healing and can improve the biological environment, including the immunological response [110]. The mechanosensitivity theory explains clinical practices, such as the use of stronger fixation or bone grafts in osteoporotic patients and the application of external stresses (e.g., massage, electro-acupuncture, ultrasound) to promote healing, especially when early movement is not possible. However, excessive fixation can lead to problems like stress shielding and periprosthetic bone resorption, reducing bone stress and causing delayed healing, non-union, or periprosthetic fractures.

Early activity provides beneficial mechanical stimuli, promoting bone and muscle growth while limiting fat accumulation. Exercise regulates osteokines, myokines, and adipokines through mechanotransduction, enhancing osteogenesis and increasing fluid flow in the bone matrix, which positively impacts bone metabolism. Thus, moderate physical activity is a clinically relevant intervention to improve bone health and combat bone aging and loss. Additionally, during fracture healing, application of non-steroidal anti-inflammatory drugs such as aspirin, activators of mechanosensitive channels, or stimulation through external stresses such as maintaining moderate mechanical activity, microneedling, and ultrasound would have beneficial effects.

#### 6.4.4 Relationship between bone metastases and weight bearing

Bone metastases are more likely in weight-bearing areas like the spine, pelvis, and proximal femur due to their rich blood supply and active metabolism. These areas experience higher mechanical stress and biomechanical loads, leading to microinjuries and continuous bone remodeling. The imbalance between bone resorption and formation alters the microenvironment, creating conditions favorable for cancer cell invasion and growth. We have developed a mechanobiological explanation for this phenomenon, the mechanosensitive protein PC1 has been found to be associated with the generation of certain tumours, integrins mediate bone metastasis in breast, prostate and lung cancers by promoting cancer cell adhesion, migration and survival, activation of YAP has also been associated with bone metastasis [111], and mechanically stimulated production of the TGF-β family such as BMP is closely associated with tumour metastasis. Osteoporosis and reduced bone mineral content increase the risk of bone metastases [112], Exercise may help to improve the quality of the bones and the organism and help to control cancer and treatment-related side effects, so mechanical stimulation may also play a protective role to some extent. The response of tumour cells to mechanical stimulation is variable, and in general mechanical stimulation reduces the proliferation of tumour cells, but tumour cells in certain areas are more likely to grow and metastasise when exposed to mechanical stimulation [113]. Exercise management for the oncology population remains a challenge that needs to be addressed at this time, and moderate, planned and individualised exercise prescriptions need to be further researched and addressed.

## 7 Conclusion and perspective

Aging impairs the mechanosensation, mechanotransduction, and mechanical effects in musculoskeletal cells, leading to decreased mechanosensitivity and accelerating bone aging. Analyzing these changes from a mechanosensitivity perspective enhances our understanding of osteoporosis and bone aging, offering insights to improve prevention and treatment strategies.

The concept of mesoscopic scales and trans mesoscopic scale transplantation therapies opens new avenues for understanding mesoscopic structures. Future research should focus on identifying common targets across mechano-pathways and developing co-activators to enhance mechanoreception and mechanotransduction, with the goal of preventing bone aging and osteoporosis. Advancements in stem cell therapy, senolytic drugs, and mechanically activated implants could provide targeted treatments for fracture healing and rehabilitation.

This multidimensional, multiscale approach offers new ideas for discovering common targets in bone aging treatment. Future research can build on this foundation to develop new therapies and drugs for bone aging and repair enhancement.

## Author contributions

All authors have read and approved the article. Zeyuan Zhang, Houchen Lyu, Yong Xie, and Licheng Zhang conceptualized and wrote the outline of the manuscript. Zeyuan Zhang and Fuming Cao searched the literature. Zeyuan Zhang, Fuming Cao, and Dingfa Liang wrote the draft of the manuscript. Meng Pan, William W Lu, Houchen Lyu, Yong Xie, Licheng Zhang and Peifu Tang reviewed and edited the manuscript.

## Data availability

No data was used for the research described in the article.

## Declaration of competing interest

The authors declare that they have no known competing financial interests or personal relationships that could have appeared to influence the work reported in this paper.
